# BIA-ALCL: Comparing the Risk Profiles of Smooth and Textured Breast Implants

**DOI:** 10.1007/s00266-023-03329-8

**Published:** 2023-03-30

**Authors:** Eric Swanson

**Affiliations:** Swanson Center, 11413 Ash St, Leawood, KS 66211 USA

*Level of Evidence V* This journal requires that authors assign a level of evidence to each article. For a full description of these Evidence-Based Medicine ratings, please refer to the Table of Contents or the online Instructions to Authors www.springer.com/00266.

In their defense of textured breast implants, Rocco et al. [1] comment, “Allergan was the market leader for decades, hence numerically it is expected that Allergan [AbbVie Inc., Lake Bluff, Ill.] implants would be present in more patients developing BIA-ALCL [Breast Implant-Associated Anaplastic Large-Cell Lymphoma], without these figures being a proof of higher risk or causality.” Of course, knowledge of the increased risk from macrotextured implants comes from data that include a denominator. Only two studies, both unreferenced by the authors, prospectively evaluate BIA-ALCL risk for Allergan Biocell breast implants [2, 3]. These studies report a risk of 1:2207 (supplemented by 4 cases diagnosed after publication) [4] and 1:355, respectively. Rocco et al. [1] do not mention the remarkable fact that not a single case of BIA-ALCL has been identified in a woman whose implant history is known with certainty to include only smooth implants [5].

In a similar letter to the editor, Mercer [6] refers to UK government guidelines that state there is not a statistically significant difference in BIA-ALCL risk between manufacturers. Rocco et al. [1] also believe that the relative risks between implants are unclear. The difference in risk is between devices and surface characteristics [5], not necessarily manufacturers of products sold in the UK, which limits the scope considerably. This association has been well-documented in studies elsewhere, particularly Australia and New Zealand [7]. According to the US Food and Drug Administration (FDA), when the manufacturer history is known, approximately 91% of world cases involved exposure to a Biocell implant [5], far greater than the percentage of women implanted with these devices, which accounted (before the ban) for only 5% of breast implants sold in the USA [8]. Indeed, the FDA banned macrotextured implants specifically because of this greater risk. Allergan has not challenged this conclusion and quickly withdrew Biocell implants from the market in response to the request from the FDA [8]. Despite evidence to the contrary, these 16 authors believe that any suggestion that patients implanted with macrotextured implants are at high risk for BIA-ALCL is misleading [1].

## Categorical Difference in Risk

The risk introduced by macrotextured implants is not relative. It is categorical [9]. Simply stated, textured implants have a risk of BIA-ALCL; smooth implants do not. For this reason, it is misleading to advise patients and plastic surgeons that the BIA-ALCL risk is “increased” when using textured implants [1], in the same way the risk of pregnancy is “increased” in biological women compared to men. The BIA-ALCL risk for implants that have a lesser degree of texturization (“microtextured”) is unknown. The risk is less than for women with macrotextured implants, but not zero [10, 11]. Mathematically, the risk of BIA-ALCL is increased infinitely when a textured device is inserted simply because the numerator becomes zero if a smooth device is used. The risk profile is not a continuum.

In 2020, the FDA issued labeling guidelines that include a boxed warning [12]. Patients are warned that breast implants are associated with BIA-ALCL, and that this cancer “occurs more commonly in patients with textured implants than smooth implants.” The first page of the Allegan boxed warning and patient checklist reiterates, “this cancer occurs more commonly in patients with textured implants than smooth implants” [13]. All women receiving implants, whether smooth or textured, are cautioned about the potentially fatal risk of BIA-ALCL. A 2021 survey shows that a boxed warning doubles the perception among women that implants are unsafe (from 29 to 58%). Half of women who read this boxed warning are less likely to consider breast implants, and more willing to consider alternatives to implants for augmentation or reconstruction [14]. These warnings do not explain that textured implants introduce a potentially fatal risk that is eliminated by choosing smooth implants. Listing this complication for both devices muddles this critical distinction. Women are likely to conclude that there is a BIA-ALCL risk for both types of implants, although it is greater for textured implants.

Unfortunately, patients may decide against breast implants, unaware that the BIA-ALCL risk is zero when using smooth implants. This confusion could have been avoided if this cancer had been originally labeled “Textured Breast Implant-Associated Anaplastic Large-Cell Lymphoma” [8], and a “TBIA-ALCL” warning issued only for this type of implant [15].

A failure to appreciate this important distinction in risk is reflected in a recent 14-author consensus statement [16] that recommends against the use of either textured or smooth implants in patients treated for BIA-ALCL, insisting on autologous reconstruction instead. The authors (incorrectly) state, “there is no definite evidence that preclude (*sic*) any association between smooth implants and the pathogenesis of BIA-ALCL” (16). This statement is remarkable because the evidence shows that smooth implants are not associated with BIA-ALCL [5], and a causal relationship has now been established between textured implants and BIA-ALCL, including (but less commonly) microtextured implants [10, 11].

Forgoing textured implants (which caused the BIA-ALCL) is universally accepted practice for secondary surgery [17], but advising against smooth replacement implants is not. Both affected and contralateral implant are removed. Women are directed toward costly, complex, and often less successful non-implant alternatives. Cosmetic breast patients become bilateral reconstructive patients. A woman who chose implants for cosmetic augmentation is now advised that she cannot have implants again and must choose fat injection, free flaps, or fat-augmented latissimus dorsi reconstructions instead [16].

## Risk/Benefit Analysis

Santanelli di Pompeo et al. [18] have shown that the risk of outpatient breast implant surgery is negligible. In their retrospective study, there were no deaths among almost 100,000 breast implant procedures. Similarly, Wixtrom et al. [19] reported a mortality of 1:72,000 for cosmetic breast outpatients. By contrast, according to the most recent figures reported by the FDA [20], BIA-ALCL carries a risk of mortality of about 5%. This diagnosis also carries a heavy morbidity [21]. Women diagnosed with BIA-ALCL require en bloc capsulectomies [21] and a metastatic workup that includes PET-CT scans. The cost of treatment can be hundreds of thousands of dollars [17]. It is a life-changing, and sometimes life-ending, event. Allergan limits its reimbursement for women who develop BIA-ALCL to US $7500 and free smooth implant replacements [22]. A release is required.

Some plastic surgeons primarily based outside the USA, where textured devices have been preferred for decades, continue to make forceful arguments in favor of textured breast implants [1, 6, 23–25]. According to the FDA, as of April 1, 2022, 1130 women worldwide have been diagnosed with BIA-ALCL and at least 59 women (5%) have died from this cancer [20]. None of these women would have died of this cancer if smooth implants were inserted instead.

Patients must be informed that explantation, with or without capsulectomy, may not eliminate risk, and the exact reduction in risk is unknown [9]. An analogy may be found in the cancer risk caused by smoking. Smoking is known to increase the risk of lung cancer. A smoker who quits still has a risk of lung cancer. However, no clinician will recommend that smokers continue the habit anyway and simply return if they develop any clinical symptoms or signs of lung cancer, or return for chest radiographs on a regular basis. Unlike women who receive smooth implants, non-smokers still have a small risk of lung cancer. This important difference only strengthens the case for not inserting textured devices, which creates an absolute difference in risk.

Advocates for textured implants often find they change their mind once they have a patient of their own diagnosed with BIA-ALCL [17]. Many surgeons who have experienced this complication in one of their patients never insert another textured device. For plastic surgeons who continue to defend textured devices, videos that include patient testimonials from the 2019 FDA Hearing are required viewing [26]. A condensed version is available online [27]. Any surgeon who can listen to women tell their stories of lives forever marred by this horrible complication, some still fighting for their lives, and continue to promote this harmful device should not be in the healing profession.

Any argument that textured implants provide a superior esthetic result is not supported by a single study using objective evaluation methods [8]. In fact, published studies show no advantage in esthetic outcome using anatomical (textured) devices [28]. Presenting a select group of cases with favorable results using textured implants [24], and no control group treated with smooth, round implants is unscientific. Experienced surgeons believe that similar results can be produced using smooth, round devices [25]. Even if there were an advantage for shaped, textured devices, any marginal esthetic benefit cannot possibly justify introducing a risk of death to a cosmetic operation.

An increased surface area caused by texturing, creating space for a heavier bacterial load, has been proposed as a factor in the development of BIA-ALCL [29] but reliable evidence is lacking. The microbiome of BIA-ALCL-affected breasts is not consistently different from contralateral control breasts [30]. Particulates and chronic inflammation are more likely to be implicated [11].

## Conflict of Interest

Rocco et al. [1] misquote a sentence from Swanson published in 2017 [15]. The actual quote reads: “This discussion leads directly to conflict of interest. Most researchers receive financial support from breast implant manufacturers, including Hu et al.” Instead of Hu et al. [31], Rocco et al. [1] substitute: “including Santanelli di Pompeo who previously reported to being affiliated with a department which received research funds from several implant manufacturers.” A comma separates the clauses. However, the reader may easily misunderstand that the sentence pertained to Dr. Santanelli di Pompeo, which is exactly the opposite of the meaning. Santanelli di Pompeo has repeatedly spoken out against textured implants [11, 18, 25], defying the industry-driven narrative [8]. Conflicted investigators defend textured implants or promote a fictitious infectious cause for BIA-ALCL rather than a device-related cause [8, 11, 32].

Loyalty to textured implants may have a financial basis. There is irony in the authors’ discussion of misleading information and financial conflicts. Mallucci, a co-author [1], recently disclosed a conflict with Polytech (Dieberg, Germany) [11, 24] and Laboratoires Sebbin (Paris, France), both textured breast implant manufacturers, and his shareholder status in B-Lite, a lightweight Polytech textured breast implant, although he claims no financial conflicts [1].

## Recognizing the Need for a Change

Rocco et al. [1] reference a study by Danilla et al. [23] that made the startling claim that smooth implants carry a greater risk of mortality than textured implants because more patients die from surgery to treat capsular contracture. Rocco et al. [1] do not reference a rebuttal [33], which discusses the flaws in this analysis. Scientifically, one must accept or reject contradictory evidence; one cannot simply ignore it. To their credit, Danilla and his colleagues are not listed among the 16 international co-authors of this new article [1].

At the 2021 World Consensus Conference on BIA-ALCL, Mallucci [24] spoke passionately in favor of textured implants. He reported that he has not encountered a BIA-ALCL case among his own patients, despite using textured implants exclusively. He considers the risk negligible. He uses the analogy of riding to work on his motorcycle as opposed to driving an automobile, reminiscent of the micromort concept [34], which suggests that the extra risk is acceptable.

Almost all plastic surgeons in the USA have transitioned to using smooth implants exclusively [17, 25]. Many surgeons have found that they do not miss anatomical, textured implants. Cordeiro [35] reports that many reconstruction patients who choose to have their Biocell implants exchanged for smooth, round devices prefer the replacements.

It is astonishing to see how staunchly textured implant advocates resist change, despite overwhelming evidence. In doing so, they align with industry and against the safety of their patients. Unfortunately, conflict of interest has a huge influence, with Allergan spending millions of dollars to fund plastic surgeons and our professional societies [8]. Many plastic surgeons will say privately (and some publicly, including Santanelli di Pompeo) that if a loved one had macrotextured devices, they would not hesitate to remove them [9].

This discussion leads to a consideration of moral hazard—the freedom to make decisions for people without having to bear the consequences. Textured implant advocates are unlikely to be sufficiently convinced of the safety of textured devices to accept responsibility for the medical treatment and financial loss to their patient if she develops BIA-ALCL. The manufacturer does not accept this responsibility either, apart from a $7500 payment. Tellingly, only smooth replacement implants are provided at no charge by Allergan for patients with BIA-ALCL [22].

## Honest Reporting of Risk and FDA Recommendations

The FDA made its initial recommendations almost 4 years ago [36]. The numerator and denominator have since changed. The most recent evidence shows that the risk of explantation or replacement with smooth devices is almost zero [18]. Recent evidence also suggests that the risk of BIA-ALCL is higher than once thought. The lifetime risk may be as high as 1:100 for Biocell implants [3, 35]. Hall-Findlay has encountered 2 cases of BIA-ALCL among only 100 cosmetic patients [37]. This information affects a risk/benefit analysis, shifting it toward implant removal or replacement [9], which may indeed reduce BIA-ALCL risk [38].

It is difficult to reconcile banning a device for causing cancer and at the same time advise patients that they do not need to have them removed [9]. This decision is for the patient to make. Women must be given an honest description of their risk of BIA-ALCL and the risk of implant removal or exchange with smooth devices. The risk of BIA-ALCL should not be understated and the risk of explantation surgery should not be exaggerated [9]. Patients should not be advised that “the FDA recommends against it,” which is untrue. The FDA does not recommend removal but does not recommend that patients keep their implants either. The FDA simply does not weigh in on this decision, preferring to leave it to the patient and her doctor to decide [36].

## “Nanotextured” Breast Implants

Over the last decade, Motiva “nanotextured” implants (Establishment Labs, Alajuela, Costa Rica) have gained popularity internationally [39, 40]. This label is a misnomer [41]. Texture is measured in micrometers, not nanometers. Smooth implants are defined as less than 10 µm of texturization and include Motiva SmoothSilk and SilkSurface implants (3.2 µm) [42]. Microtextured implant surfaces have 10–50 µm of roughness, and macrotextured implants feature surfaces with greater than 50 µm of texture. Not surprisingly, smooth implants and Motiva SilkSurface implants also have the lowest surface areas among implants (80–100 mm^2^), not much greater than a flat surface (79 mm^2^) [42].

Such limited texturing is not known to cause tissue adherence. Clinically, these implants resemble smooth devices. Round devices are preferred [39, 40]. Motiva markets its shaped breast implant with tabs for tissue fixation as the only “smooth” anatomic breast implants currently available [43]. The word “nanotextured” is not to be found on the website. Motiva implants are not approved by the FDA and are not available in the USA [43]. Many plastic surgeons consider nanotextured implants to be essentially smooth implants [40, 44].

Implants manufactured by Motiva are marketed as a sweet spot between smooth and conventional textured implants [39]. Although a “nanotextured” surface is promoted to reduce the risk of capsular contracture [39, 43], a recent retrospective study found no advantage for these products compared with traditional textured implants made by other manufacturers [39]. No controlled studies are available comparing nanotextured and smooth implants [41]. Financial conflict of interest is an issue. Most studies of Motiva implants are authored by plastic surgeons with a financial conflict [39, 40, 42].

“Smooth” would seem to represent a more accurate label for these products. If no benefit from very light texturing can be reliably demonstrated, why not simply implant smooth devices exclusively? It is time to relegate textured implants (and the name) to an unfortunate chapter in the history of plastic surgery. A course correction is needed, and, for many plastic surgeons, it has already taken place. Limiting the BIA-ALCL warning to textured implants and a recommendation to transition to smooth implants is not new. The referenced original 2017 publication making these recommendations [15] concluded, “The industry can adapt, as it has in the past. The problem, and its resolution, could not be clearer.”
